# Understanding the Paradigm of Molecular-Network Conformations in Nanostructured Se-Rich Arsenoselenides As_x_Se_100−x_ (x < 10)

**DOI:** 10.3390/molecules30163380

**Published:** 2025-08-14

**Authors:** Oleh Shpotyuk, Zdenka Lukáčová Bujňáková, Yaroslav Shpotyuk, Andriy Kovalskiy

**Affiliations:** 1 Institute of Physics, Jan Dlugosz University in Częstochowa, 13/15, al. Armii Krajowej, 42-200 Częstochowa, Poland; 2 O.G. Vlokh Institute of Physical Optics, Ivan Franko National University of Lviv, 23, Dragomanov Str., 79005 Lviv, Ukraine; 3 Institute of Geotechnics, Slovak Academy of Sciences, 45, Watsonova Str., 04001 Košice, Slovakia; bujnakova@saske.sk; 4 Department of Sensor and Semiconductor Electronics, Ivan Franko National University of Lviv, 107, Tarnavskoho Str., 79017 Lviv, Ukraine; yashpotyuk@gmail.com; 5 Institute of Physics, University of Rzeszow, 1, Pigonia Str., 35-959 Rzeszow, Poland; 6 Department of Physics, Engineering and Astronomy, Austin Peay State University, Clarksville, TN 37044, USA; kovalskiya@apsu.edu

**Keywords:** molecular-forming clusters, network-forming clusters, arsenoselenides, nanomilling, amorphization, medium-range structure, X-ray diffraction, cluster modelling

## Abstract

The paradigm of molecular-network conformations in Se-rich glassy arsenoselenides As_x_Se_100−x_ compositionally approaching pure Se (x < 10) is considered, employing comprehensive XRD analysis of diffuse peak-halos and nanocrystalline reflections from the known Se polymorphs in their XRD patterns. Within a modified microcrystalline model, the changes with growing Se content in these alloys are interpreted in terms of suppression in intermediate range ordering due to shifting to high diffraction angles and a narrowed FSDP (first sharp diffraction peak)-related diffuse peak-halo, accompanied by enhancement in extended range ordering due to a shift to low diffraction angles and a broadened SSDP (second sharp diffraction peak)-related peak-halo. Overlapping of these peak-halos is enhanced in Se-rich alloys, tending towards unified FSDP-SSDP-related halos with characteristic doublet asymmetry due to the remnants of nanocrystalline trigonal *t*-Se. Drastic enhancement of the crystallization processes related to the trigonal *t*-Se phase is a principal feature of nanostructurization effects in Se-rich glassy arsenoselenides driven by nanomilling. The nanostructurization response in these alloys is revealed as a fragmentation impact on the correlation length of the FSDP-responsible entities, accompanied by an agglomeration impact on the correlation length of the SSDP-responsible entities. The FSDP- and SSDP-related diffuse peak-halos become more distinguishable in the XRD patterning of nanostructured arsenoselenides, being associated with other contributions from crystalline remnants, such as those expected in transition to glassy arsenoselenides with higher Se content. An irregular sequence of randomly distributed *cis*- and *trans*-configurated multiatomic Se linkages is visualized by ab initio quantum-chemical modeling of Se_n_ chain- and ring-like conformations. The most critical point of molecular-network disproportionality analysis in the examined arsenoselenide As_x_Se_100−x_ glassy alloys obeying the chain-crossing model corresponds to x = 7 (equivalent to 93 at. % of Se in the binary As-Se system), as an equilibrium point between mixed *cis*-*trans*-configurated Se_7_ chains and exceptionally *cis*-configurated molecular Se_8_ rings. At the basis of developed models, the paradigm of thermodynamically stable molecular-network conformations in the nanostructured Se-rich arsenoselenides As_x_Se_100−x_ (*x* < 10) is surely resolved in favor of chain-like network-forming conformations composed of mixed *cis*-*trans*-configurated network-forming multiatomic Se fragments.

## 1. Introduction

Binary arsenoselenide alloys of the canonical As_x_Se_100−x_ system compose an important class of amorphous substances which can be easily stabilized in a structurally disordered state by a conventional melt-quenching route within a broad domain around stoichiometry corresponding to arsenic triselenide As_2_Se_3_ (x = 40), stretching downwards to under-stoichiometric Se-rich alloys terminated by amorphous a-Se (0 ≤ x < 40) and upwards to over-stoichiometric As-rich alloys having up to ~70–75 at. % of As [1,2]. During past decades, these disordered materials have been uniquely functionalized at the nanoscale by advanced nanostructurization technologies such as high-energy mechanical milling (nanomilling) [3,4], attracting great attention in the glass science community in view of their promising perspectives in photonics [5,6,7] and biomedicine [8,9,10].

From a contemporary glass manufacturer’s viewpoint, especially attractive possibilities have been ascribed to over-stoichiometric arsenoselenides As_x_Se_100−x_ (x > 40), having molecular-network conformations based on thioarsenide-type As_4_Se_n_ species (0 < n < 6) stabilized in a saturated As-Se covalent network [11,12,13]. Tuning the fraction of molecular and network species in these alloys by available nanostructurization approaches such as nanomilling, the functionality of these alloys was essentially modified, finalizing in a new family of special chalcogenide glasses with improved thermodynamic heat-transfer responses [12,13]. In contrast, the under-stoichiometric As_x_Se_100−x_ alloys (x < 40) exhibit conformations possessing layered- and chain-type networks (due to long Se chains bridging AsSe_3/2_ pyramids), which are almost insensitive to post-preparation modification [11]. The only exception is expected for Se-rich arsenoselenides As_x_Se_100−x_ approaching Se (0 ≤ x < ~10), where some modification possibilities (albeit more hidden as compared with those in over-stoichiometric As_x_Se_100−x_ alloys, x > 40 [11,12,13]) are still possible due to variations in *cis*- and *trans*-configurated species characteristic for Se polymorphs [14,15]. Thus, molecular ring-like species (such as Se_8_ or Se_6_), which consist exceptionally of *cis*-configurated five-membered Se linkages typical for α- and β-monoclinic Se [15,16,17,18,19], can be stabilized in amorphous a-Se in addition to spiral species consisting of *trans*-configurated helical Se_n_ fragments typical for trigonal *t*-Se [15,20].

The paradigm of molecular-network conformations emerged from the disordered chain molecule model of Misawa and Suzuki [21,22] explaining thermally activated ring-to-chain transformations in liquid Se. In turn, this model was developed as an extension of the rotational isometric state model of Semlyen [23], justifying the preference of cyclic polymerization over linear polymerization in some chalcogen species like Se chains in view of the asymmetric intramolecular rotating potential between them. The potential energy minimum ascribed to five Se atoms in a so-called *cis*-configurated linkage was 0.62 kcal/mol deeper than that ascribed to *trans*-configurated Se atoms because of attractive Van der Waals interaction between the first and fifth Se atoms. Therefore, the structural arrangement of Se linkages was assumed to be more complicated in liquid-quenched a-Se. According to Lucovsky et al. [24], distorted Se_n_ chains forming an amorphous a-Se network consist of an irregular sequence of randomly distributed *cis*- and *trans*-configurated fragments acting as isolated species, which prevail over cyclic multiatomic Se species (such as ring-like Se_8_ or Se_6_ molecules).

This paradigm has been dramatically revealed in application to nanostructured As_x_Se_100−x_ alloys approaching pure Se (x = 0), which could be exemplified by the pressure and milling-induced effects in a-Se. Thus, in the early 1990s, Keiji Tanaka [25] showed that with increasing pressure applied under hydrostatic compression (up to ~120 kbar), the diffuse peak-halo at ~2 Å^−1^ in the X-ray diffraction (XRD) pattern of a-Se (undergoing amorphous I-to-amorphous II transition) was gradually shifted to higher diffraction angles with an increase in the peak height and decrease in the peak width, showing slight hysteresis under further depressurization. Under applied pressure above ~120 kbar, contraction of interchain distance in a-Se was finalized in transition to a hexagonal *t*-Se phase consisting of helical Se_n_ chains (amorphous-to-crystalline phase transition). With increased pressure applied to *t*-Se (above ~140 kbar), the weakening of intrachain bonds and strengthening of interchain bonds led to a hexagonal-to-monoclinic transition (crystalline I-to-crystalline II transition), finishing by the formation of Se_8_ molecular species [25].

Similar transformations through a number of amorphous and crystalline phases were also activated in melt-quenched a-Se subjected to nanomilling. In the late 1990s, complete amorphization of *t*-Se into an as-milled phase possessing a lower crystallization temperature due to destroyed bonding between chain molecules caused by mechanical strains and defects was reported by Fukunaga et al. [26]. Guo and Lu [27] observed an amorphous–nanocrystalline–amorphous phase transition in a-Se subjected to grinding. Upon initial milling, the as-quenched amorphous phase composed of predominant Se_8_ rings and Se_n_ chains (a-Se^I^) was crystallized completely in a nanocrystalline *t*-Se phase. Under further milling, the crystalline reflexes of *t*-Se were gradually decreased in the XRD patterns at a cost of abnormally growing diffuse peak-halos ascribed to an as-milled amorphous phase composed of Se_n_ chains (a-Se^II^) and crystalline reflexes of a α-Se phase composed of Se_8_ ring-like molecules. Because of the destroyed interaction between these chains, the as-milled a-Se^II^ phase shows a lowering in the crystallization temperature and enthalpy as compared with the as-quenched amorphous phase (a-Se^I^). Thus, the microstructure of a-Se is essentially dependent on the pre-milling state of the substance undergoing nanostructurization.

As was shown in the early 2000s by J.C. de Lima with co-workers [28], the amorphous structure of melt-quenched a-Se can be described as consisting of slightly distorted Se_8_ rings along with an occasional ring open sufficiently to develop a local trigonal symmetry or a few greatly deformed Se_n_ chains, while the atomic arrangement in the milled sample consists of Se_n_ chain molecules only. Under prolonged aging-initiated amorphous Se_n_ chain-to-trigonal phase transformations, the structure of a-Se derived by milling consists of Se_8_ rings embedded in an environment close to nanocrystalline trigonal *t*-Se (therefore, more energy is needed to promote Se crystallization in this state).

Nearly a decade later, this finding was questioned by Boolchand and co-workers [29], distinguishing between (i) melt-quenched bulk Se glass subjected to long-term 8-year aging and (ii) powder of the 8-year aged glass subjected to 2-week laboratory ambient storage. The Se_8_ rings were found to appear in the short-term aged Se powder, resulting in crystallization of the monoclinic Se phase, while only nanocrystalline reflexes assigned to *t*-Se overlapped with diffuse peak-halos of the amorphous phase were found in the XRD patterns of long-term aged Se glass. Despite an obvious difference in the pre-history of the examined substances (melt-quenched bulk a-Se from one side and powdered a-Se with admixture of nanocrystalline *t*-Se from the other side), the authors [29] evidently speculated on the uniqueness of the molecular origin of physical aging in bulk g-Se. In their interpretation based on frequency-free deconvolution of bond-stretching vibrational modes in the collected Raman spectra, the increase in the 260 cm^−1^ peak intensity in addition to its low-frequency shifting was ascribed in the aged g-Se powder to an increased fraction of Se_8_ molecules decoupled from a glass backbone, instead of a decrease in this peak, as could be expected from a reduction in the FWHM of the overall three-modal peak. This conclusion signalizes on an evident artifact of the fitting procedure applied in [29]. Nevertheless, assuming the possibility of such transformations (as in pure g-Se) for Se-rich Ge-Se glasses, the authors [30] come to a very controversial conclusion on the super-flexible phase in these glasses, which could appear due to decoupling and further ‘disappearing’ of Se_8_ ring-like molecules from a glass network subjected to physical aging.

In the above cases, the paradigm of molecular-network conformations in Se-rich glassy arsenoselenides has been considered, regardless of the analysis of diffuse peak-halos and nanocrystalline reflexes arising from Se polymorphs in their XRD patterning. The objective of this research is a comprehensive XRD analysis in searching for the direct medium-range structural response on nanomilling-driven transformations in glassy g-As_x_Se_100−x_ alloys compositionally approaching pure Se (x < 10). As in the case of over-stoichiometric molecular-network arsenoselenides As_x_Se_100−x_ (x > 40) [11,12,13], an insight into nanostructurization grounded on a disproportionality analysis of *cis*- and *trans*-configurated multiatomic Se linkages will be developed for Se-rich glassy arsenoselenides, employing an ab initio quantum-chemical modeling approach.

## 2. Results and Discussion

### 2.1. Compositional Changes in the XRD Patterning in Se-Rich As_x_Se_100−x_ Alloys (x < 10)

The medium-range structure related to the XRD patterning in arsenoselenide alloys was adequately parameterized via employing a modified microcrystalline model [11,12,13]), assuming decomposition of the XRD patterns on a few peak-halos, three of which (the first sharp diffraction peak, FSDP, the second sharp diffraction peak, SSDP, and the third sharp diffraction peak, TDP) were the principal ones (meaning their complete reproducibility in the structure factor determination) [31,32,33,34]. These parameters for some of the glassy g-As_x_Se_100−x_ alloys are given in our preliminary research [14].

**Figure 1:**
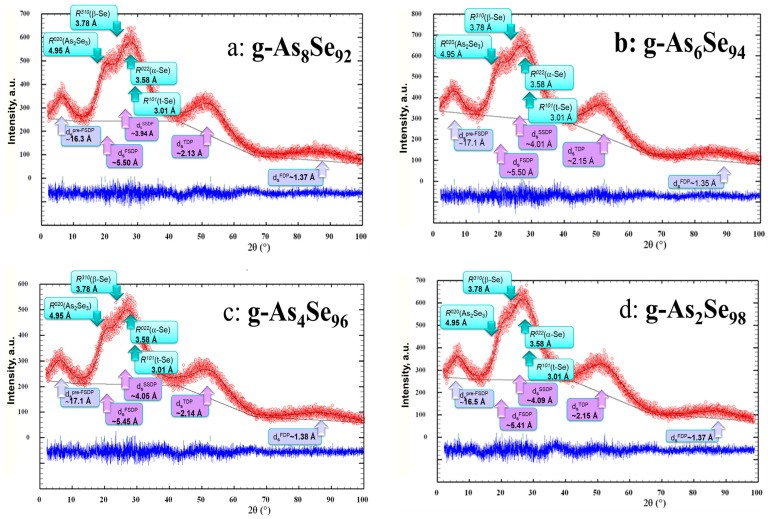
Experimental (red points) and calculated (black solid line) XRD profiles collected from some Se-rich glassy arsenoselenides, g-As_8_Se_92_ (**a**), g-As_6_Se_94_ (**b**), g-As_4_Se_96_ (**c**), and g-As_2_Se_98_ (**d**), showing arrangement of diffuse peak-halos in respect to prominent inter-planar correlations ascribed to trigonal *t*-Se and monoclinic As_2_Se_3_, α-Se, and β-Se, highlighted by bright-blue arrows. The difference curve is reproduced at the bottom in a blue color. The inter-molecular correlations corresponding to diffuse peak-halos are denoted at the bottom by purple-shadowed arrows.

The XRD patterns of Se-rich g-As_x_Se_100−x_ alloys approaching ‘pure’ Se (g-As_8_Se_92_, g-As_6_Se_94_, g-As_4_Se_96_, and g-As_2_Se_98_) collected in a region up to 2*θ*~100° are shown in Figure 1. For clarity, these XRD patterns are reproduced in comparison with the most prominent Bragg-diffraction reflexes ascribed to monoclinic As_2_Se_3_ [35,36] and Se allotropes such as trigonal *t*-Se [15,20], monoclinic α-Se [16,17], and monoclinic β-Se [18,19].

As can be inferred from parameterization of the collected XRD patterns reproduced in Figure 1, with an increase in Se content in these alloys the FSDP-responsible peak-halo positioned at 2*θ*~(20–21)° (corresponding to scattering vectors *Q*_1_ = *Q^FSDP^*~(1.4–1.5) Å^−1^ and inter-atomic Ehrenfest-diffraction distances d_s_~(5.1–5.5) Å) shifts towards high diffraction angles and scattering vectors approaching the SSDP-responsible diffuse peak-halo at 2*θ*~(26–29)° (*viz*. *Q*_2_ = *Q^SSDP^*~(2.0–1.9) Å^−1^ and d_s_~(3.9–4.1) Å), which moves in an opposite side towards low diffraction angles (and smaller *Q*_2_). These compositional changes lead to interatomic distances *d* in the overall FSDP-SSDP-related diffuse peak-halo, which are in excellent agreement with mean interatomic spacing *d_s_^m^* derived from the macroscopic densities of the glasses [14]. With increased Se content, both diffuse peak-halos (the FSDP and SSDP) overlap, transforming into an overall peak-halo showing a characteristic doublet shape with some asymmetry in magnitude at the higher diffraction angles 2*θ*, this trend being well agreed with by the data of other authors [37,38,39,40].

**Figure 2:**
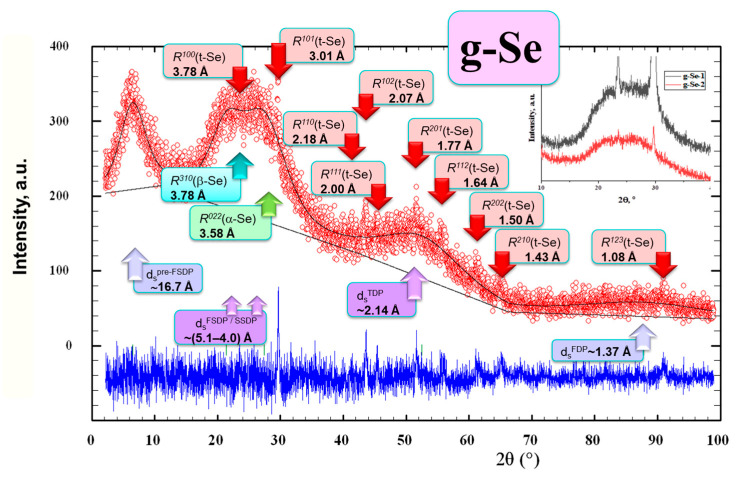
Experimental (red points) and calculated (black line) XRD profile in a melt-quenched sample of Se showing the arrangement of diffuse peak-halos overlapped with the most prominent inter-planar correlations in trigonal *t*-Se (red arrows), the difference curve being shown at the bottom in a bright-blue color. The intermolecular correlations positioning the diffuse peak-halos are denoted at the bottom by purple-shadowed arrows. The strongest inter-planar correlations from the monoclinic arrangement of Se_8_ molecules in α-Se and β-Se are respectively denoted by green and blue arrows. The insert shows the emergence of a doublet structure in the unified FSDP-SSDP-related peak-halo (due to diffraction reflections (100) and (101) ascribed to *t*-Se) in two g-Se samples.

Noteworthy, no considerable changes were observed in the higher order diffuse peak-halos responsible for the third diffraction peak (TDP, positioned at 2*θ*~(50–55)°, *viz*. *Q*_3_ = *Q^TDP^*~(3.60–3.62) Å^−1^ and d_s_~(2.1–2.2) Å) and the fourth diffraction peak (FDP, positioned at 2*θ*~(87–88)°, *viz*. *Q*_4_ = *Q^FDP^*~(5.6–5.7) Å^−1^ and d_s_~(1.35–1.37) Å), testifying in favor of negligible compositional effects at the shortest inter-atomic distances in the alloys [14]. The same concerns the pre-FSDP-related peak-halo positioned in Figure 1 and Figure 2 at 2*θ*~(6.4–6.6)° (*viz*. *Q*_0_ = *Q^pre-FSDP^*~(0.45–0.47) Å^−1^ and d_s_~(16–17) Å), which reflects prolonged inter-atomic correlations in the alloys stretching at a length scale above ~(16–17) Å [39].

The XRD pattern of a ‘pure’ g-Se specimen showing diffuse peak-halos overlapped with most prominent inter-planar Bragg-diffraction correlations ascribed to Se_n_ chains in *t*-Se and crown-like Se_8_ molecules in monoclinic α-Se and β-Se is depicted in Figure 2. It is seen that the doublet structure of the first diffuse peak-halo in this g-Se specimen at 2*θ*~26.1° (*viz*. *Q*~1.84 Å^−1^ and d_s_~4.20 Å) is revealed in two sub-peak-halos at 2*θ*~21.4° (*Q*~1.51 Å^−1^, d_s_~5.11 Å) and 2*θ*~27.4° (*Q*~1.94 Å^−1^, d_s_~4.00 Å), which emerge from two inter-planar correlations in nanostructured trigonal *t*-Se, in part, *R*^100^ = 3.7812 Å at 2*θ* = 23.509° (*I* = 43.9%) and *R*^101^ = 3.0056 Å at 2*θ* = 29.699° (*I* = 100%) [15,20].

Careful inspection of the XRD pattern shown in Figure 2, along with the XRD patterns of g-Se with different amounts of *t*-Se (insert in right-upper corner of Figure 2), confirms the doublet structure of this peak-halo is indeed connected with the most intensive peaks of nanosized *t*-Se. Similar specificity in the XRD patterns of melt-quenched Se was pointed out by Keiji Tanaka in his famous research on pressurization in a-Se [25].

Primarily and most consistently, the above findings mean that the characteristic sizes of structural species responsible for intermediate-range ordering (IRO, defined merely by the FSDP [31]) and extended-range ordering (ERO, defined merely by the SSDP [32]) are similar in the examined Se-rich glassy arsenoselenides g-As_x_Se_100−x_ (x < 10). The governing trend in these alloys at increasing Se content is suppressed IRO due to a high-shifted *Q*_1_ and narrowing of the FSDP-related diffuse peak-halo, accompanied by enhanced ERO due to a low-shifted *Q*_2_ and broadening of the SSDP-related diffuse peak-halo. Overlapping of the above peak-halos is essentially enhanced, tending to a unified FSDP-SSDP-related peak-halo with a characteristic doublet asymmetry for remnants of the trigonal arrangement of Se_n_ chains, these changes being most clearly revealed in ‘pure’ g-Se (see Figure 2).

Within a modified microcrystalline model [11,12,13], the XRD patterning in glassy chalcogenides can be adequately interpreted, assuming superimposed responses from quasi-crystalline remnants possessing the most prominent inter-planar and inter-atomic (inter-molecular) correlations, respectively revealed in Bragg and Ehrenfest diffraction.

The inter-planar correlations from remnants of the monoclinic As_2_Se_3_ phase [35,36] prevail by Bragg-diffraction line (020) at high diffraction angles of the FSDP-related peak-halo (Figure 1 and Figure 2), corresponding to inter-layer spacing *R*^020^(As_2_Se_3_)~4.95 Å (*I* = 91.2%). It seems quite reasonable that a reduction in these correlations in g-As_x_Se_100−x_ with growing Se content results in a shifting of the FSDP position to high diffraction angles, since other reflexes from this phase contribute to the high-angular wing of the SSDP.

Despite that the structure of a-Se is definitely not quasi-crystalline [41], the remnants of Se allotropes contribute to the XRD patterning in Se-rich arsenoselenides, especially due to correlations inside a -4 Å sphere. Indeed, the most prominent Bragg-diffraction reflexes of a trigonal allotrope composed of helical Se_n_ chains in preferential *trans*-conformations (*R*^100^(*t*-Se) = 3.7812 Å and *R*^101^(*t*-Se) = 3.0056 Å) fit in the angular domain of the FSDP-SSDP-related peak-halo, defining its asymmetric doublet structure (see Figure 1 and Figure 2). The strongest Bragg-diffraction reflexes of monoclinic α-Se and monoclinic β-Se possessing different arrangements of eight-membered *cis*-configurated crown-like Se_8_ molecules (with *R*^022^(α-Se) = 3.58 Å and *R*^310^(β-Se) = 3.78 Å) [15,16,17,18,19] contribute to the right side of the unified FSDP-SSDP-related peak-halo.

Similar conclusions follow from the Ehrenfest-diffraction responses of Se allotropes. Thus, in *t*-Se [15,20,25], each Se atom has four neighbors on three adjacent chains so that the first-nearest interchain distance contributing to the Ehrenfest diffraction approaches ~3.44 Å. In a glassy state, this distance is expanded to a Van der Waals distance of 3.72 Å coinciding with the second-nearest intrachain bond length, whereas the first-nearest intra-chain bond length is 2.38 Å and the second-nearest interchain distance is ~4.37 Å [25]. In contrast, both monoclinic Se forms possess higher inter-molecular distances [15,16,17,18,19,20]. In α-Se [16,17], they fit in the (3.53–3.99) Å range, being averaged around ~3.80 Å, while in β-Se, they fit in the (3.48–3.96) Å range, being averaged around ~3.78 Å [19]. Since these distances in the examined glassy arsenoselenides are commensurable with the SSDP position, the transition to amorphous structures having more ring-like molecular forms shifts the SSDP-responsible peak-halo towards lower diffraction angles 2*θ* corresponding to higher inter-molecular distances, contributing to the Ehrenfest diffraction. More prolonged but weaker inter-chain correlations in *t*-Se approaching ~5.70 Å [42], contributing rather to the FSDP, can be accepted as a signature of *trans*-configurated Se atoms stabilized within Se_n_ chains (since *cis*-configurated Se atoms within ring entities have no distinct correlations in the ~(5–8) Å range [15,42]). Hence, disappearance of this inter-molecular distance in glassy arsenoselenides enriched in *cis*-configurated Se atoms corresponds to the vanishing of the FSDP-responsible diffuse peak-halo, as shown in Figure 1.

### 2.2. XRD Patterning of Nanostructurization Response in Se-Rich As_x_Se_100−x_ Alloys (x < 10)

As was reported by Shpotyuk and co-workers [11,12,13], the primary effect of nanostructurization in over-stoichiometric arsenoselenides As_x_Se_100−x_ (x > 40), which possess mixed molecular-network conformations due to thioarsenide-type As_4_Se_n_ molecular entities dissolved in a fully saturated As-Se covalent-bond backbone, is the destruction of these entities followed by their insertion in the newly polymerized network. Such molecular-to-network transformations are accompanied by iso-typical changes in the FSDP and SSDP responsible for diffuse peak-halos, resulting in high-angular shifting in the diffuse peak-halo position and broadening in the diffuse peak-halo width. In such a case, unprecedently strong molecular-to-network transformations in these arsenoselenides result from notable changes in the nearest-neighbor interactions of the constituent atoms forming homo- and heteronuclear covalent bonding within a modified glassy backbone.

Similar effects (albeit more depressed in view of one preferential type of covalent bonding) could be expected in under-stoichiometric g-As_x_Se_100−x_ alloys approaching pure Se (x < 10), where greatly deformed Se_n_ chains (consisting of *trans*-configurated Se linkages facilitating the formation of a 1D chain-like network) are combined with distorted Se_8_ molecular species consisting exceptionally of *cis*-configurated Se linkages [14]. However, in Se-rich arsenoselenides compositionally approaching ‘pure’ Se, such transformations occur within the same homonuclear bonding (with homonuclear Se-Se bond lengths averaged around ~2.3 Å, mean atomic coordination approaching ~2.0, and valency -Se-**Se**-Se- bond angle close to ~105° [15,43]). In such a case, diversity in mixed molecular-network conformations emerges only from differences in the dihedral angles of the *cis*- and *trans*-configurated Se linkages characteristic of amorphous and crystalline Se polymorphs, which are ~70–100° for a-Se [43], ~100.6° for *t*-Se [15], and 101.0° for α/β-Se [15].

Thus, the notable differences between nanostructurization in these arsenoselenide alloys are as follows: (i) in over-stoichiometric As_x_Se_100−x_ alloys (x > 40), molecular species (such as As_4_Se_n_ thioarsenide-type molecules) decoupled from the network facilitate the stabilization of the close-to-stoichiometry (As_2_Se_3_) network, which is energetically most favorable, preferring heteronuclear As-Se covalent bonding, whereas (ii) in under-stoichiometric As_x_Se_100−x_ alloys approaching Se (x < 10), molecular species (such as Se_8_ rings) decoupled from the network do not significantly change its energetic bonding, which is invariant, preferring homonuclear Se-Se covalent bonds.

**Figure 3:**
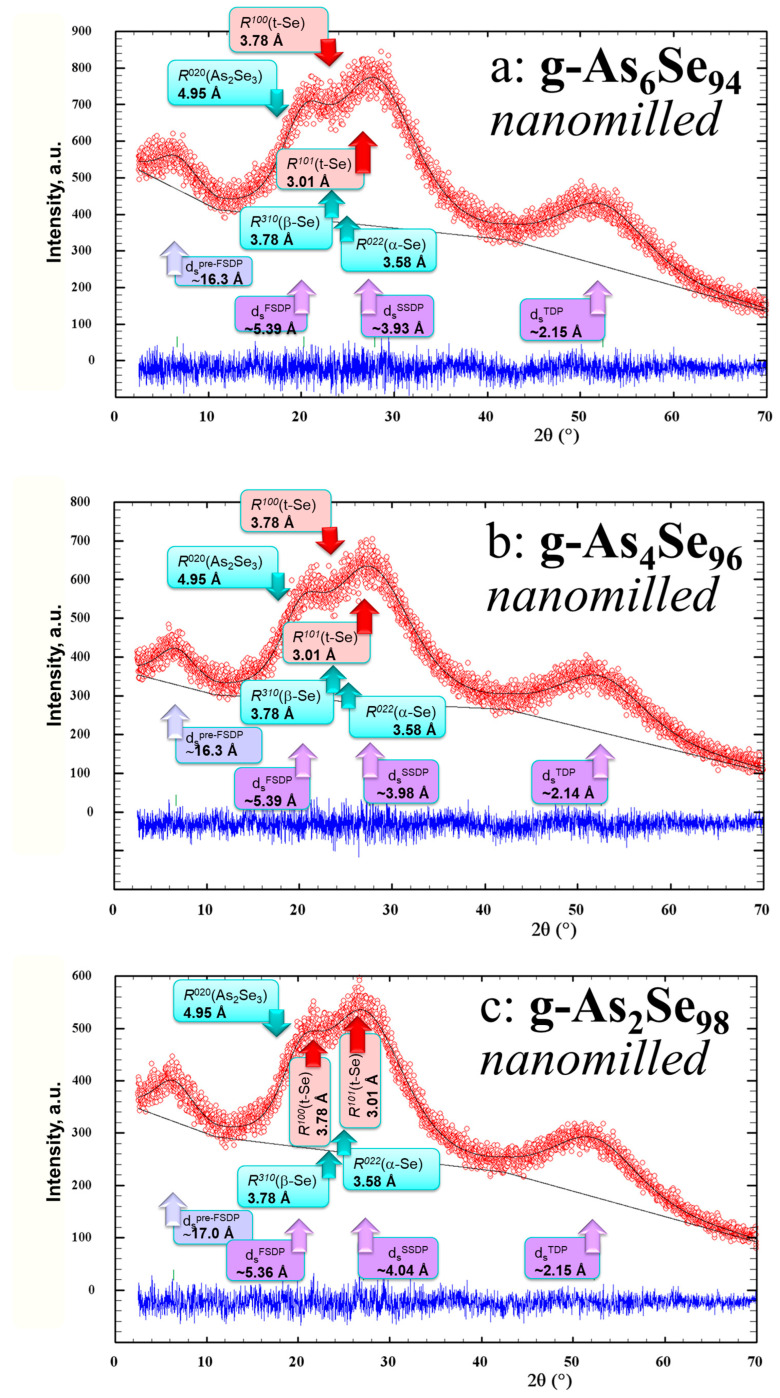
Evolution of experimental (red points) and calculated (black solid line) XRD profiles in nanomilled glassy arsenoselenides with growing Se content, As_6_Se_94_ (**a**), As_4_Se_96_ (**b**), and As_2_Se_98_ (**c**), the difference being presented at the bottom in blue color. Three principal peak-halos (highlighted by purple-shadowed arrows) are overlapped with prominent reflections in trigonal *t*-Se (highlighted by red arrows) and monoclinic As_2_Se_3_, α-Se, and β-Se (highlighted by bright-blue arrows).

The XRD patterns collected for the examined arsenoselenides approaching pure Se such as As_6_Se_94_, As_4_Se_96_, and As_2_Se_98_ subjected to nanomilling are depicted in Figure 3. To clarify the origin of crystallization processes in these samples, we reproduce these XRD patterns in comparison with two most prominent Bragg-diffraction lines characteristic of *t*-Se corresponding to inter-planar distances *R*^100^(*t*-Se) = 3.7812 Å (2*θ* = 23.509°, *I* = 43.9%) and *R*^101^(*t*-Se) = 3.0056 Å (2*θ* = 29.699°, *I* = 100%) [15,20]. In our analysis, we also consider the strongest inter-planar correlations in these samples expected from monoclinic phases, such as *R*^020^(As_2_Se_3_) = 4.9519 Å (2*θ* = 17.898°, *I* = 91.9%) [35,36], *R*^022^(α-Se) = 3.5757 Å (2*θ* = 24.880°, *I* = 100%) [16,17], and *R*^310^(β-Se) = 3.7790 Å (2*θ* = 23.522°, *I* = 100%) [18,19].

As it follows from Figure 3, nanomilling-driven crystallization of the *t*-Se phase is the primary crystallization process, drastically enhanced in the examined arsenoselenides enriched in Se content. Indeed, the most prominent Bragg-diffraction lines ascribed to nanocrystalline inclusions of the *t*-Se phase *R*^100^(*t*-Se) = 3.7812 Å and *R*^101^(*t*-Se) = 3.0056 Å clearly revealed at the background of the overlapped FSDP-SSDP-related peak-halo are practically invisible after nanomilling in the XRD patterning of g-As_6_Se_94_ (see Figure 3a), but slightly visible in the XRD pattern of more Se-enriched g-As_4_Se_96_ sample (see Figure 3b), getting to be well observable in the XRD patterning of g-As_2_Se_98_ sample (Figure 3c).

**Figure 4:**
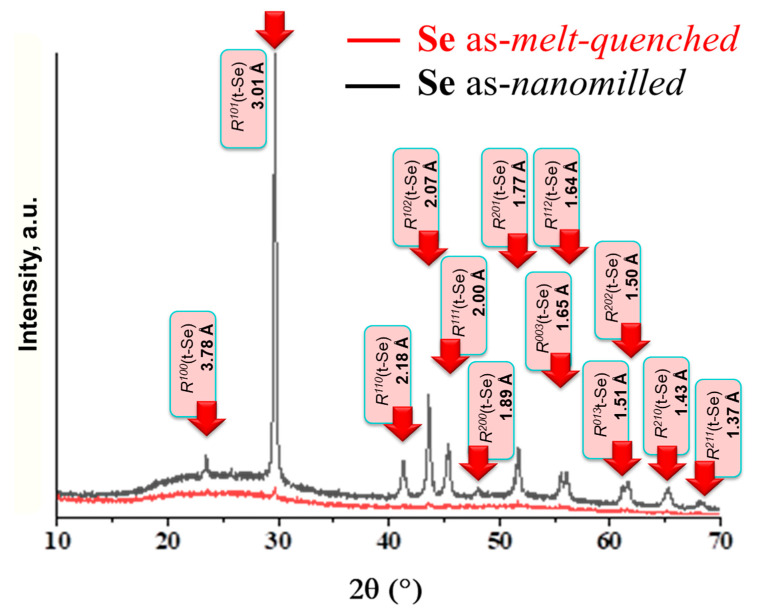
Comparative presentation of the XRD profiles collected for melt-quenching derived Se specimens before (red curve) and after nanomilling (black curve). The broadened inter-planar Bragg-diffraction peaks ascribed to *t*-Se (the JCPDS card No. 73-0465 [20]) become evidently dominated at the background of principal diffuse peak-halos in Se samples subjected to milling.

Specifically, in the melt-quenching-derived pure Se sample, which was rather glassy-crystalline before nanomilling in view of extractions of the trigonal *t*-Se phase, almost complete crystallization of this phase prevails over amorphization (re-amorphization), defined by changes in diffuse peak-halos, as this follows from the XRD patterns reproduced in Figure 4.

Thus, drastic enhancement of crystallization processes related to the trigonal *t*-Se phase is a principal feature of nanostructurization-driven effects in the examined As_x_Se_100−x_ alloys with highly growing Se content. Specifically, changes in the medium-range structure of these alloys are defined by the arrangement of the FSDP- and SSDP-related diffuse peak-halos in their XRD patterns collected before (Figure 1) and after nanomilling (Figure 3). As it follows from the comparison of these XRD patterns, under milling-induced nanostructurization in these alloys, we deal with enhancement of IRO due to a slightly high-shifted *Q*_1_ but also an essentially broadened FSDP-related diffuse peak-halo, counterbalanced by depression of ERO due to a high-shifted *Q*_2_ but also an essentially narrowed SSDP-related diffuse peak-halo. This means that nanomilling causes a fragmentation impact on correlation length *L* of the FSDP-responsible entities, but an opposite agglomeration impact on the SSDP-responsible entities. As a result, the FSDP- and SSDP-responsible diffuse peak-halos become more distinguishable in the XRD patterning of nanomilled arsenoselenides (purely nanostructurization trend), being associated with other contributions from quasi-crystalline remnants, such as those expected in the transition to more Se-rich As_x_Se_100−x_ alloys with higher Se content (purely compositional trend).

### 2.3. Computational Insight on Nanostructurization in Se-Rich As_x_Se_100−x_ Alloys (x < 10)

The microstructure of Se-rich arsenoselenides As_x_Se_100−x_ (x < 10) can be interpreted in terms of a disordered chain- and ring-like molecule model (see, e.g., [21,22,28,43]), in which slightly distorted Se chains consisting of irregular sequences of randomly distributed *cis*- and *trans*-configurated five-membered Se linkages prevail in an amorphous backbone over ring molecules (such as Se_8_) composed of *cis*-configurated Se linkages. Acting essentially as isolated species, such chain- and ring-like molecules possess much weaker intermolecular interactions in an amorphous state as compared with crystalline.

Within this model, nanostructurization in the arsenoselenides can be considered as resultant of *quasi molecular*-*to*-*network* transition originated from changes in the fraction of Se atoms forming *cis*- and *trans*-configurated linkages [14]. Accepting the positioning of the most prominent Bragg- and Ehrenfest-diffraction reflexes in Se polymorphs consisting of Se chains (as in *t*-Se [20]) and/or ring Se_8_ molecules (as in α-Se [16,17] or β-Se [18,19]), the governing compositional trend in these alloys becomes understandable. The overlapping of the FSDP- and SSDP-responsible peak-halos in their XRD patterns, enhanced at a higher content of two-fold coordinated Se atoms, can be interpreted as an effect corresponding to the enriched molecular character of long Se_n_ chains bridging three-fold coordinated AsSe_3/2_ units due to the higher fraction of *cis*-configurated fragments within these chains.

Assuming a chain-crossing model for the Se-rich glassy arsenoselenides under research (which means a fully homogenized covalent bond matrix with uniform distribution of constituents [1,2]), the following transformation could be expected:

>As–Se_n_–As< → >As–Se–As< + Se_n−1_, (1)

where >As–Se–As< corresponds to one leg of so-called corner-shared AsSe_3/2_ pyramids (which is known to be the most energetically favorable in binary arsenoselenides [13]), and the Se_n−1_ cluster adopts the most favorable conformations of Se atoms branching these units.

Let us examine such conformations in the highly Se-rich arsenoselenide alloys based on self-consistent ring-like Se_n_^ring^ molecules (alternatively, MFCs, molecular-forming clusters) and their chain-like derivatives formed by breaking in Se atom position Se_n_^chain^ (alternatively, NFCs, network-forming clusters) employing ab initio quantum-chemical modeling in terms of CINCA [44,45].

**Figure 5:**
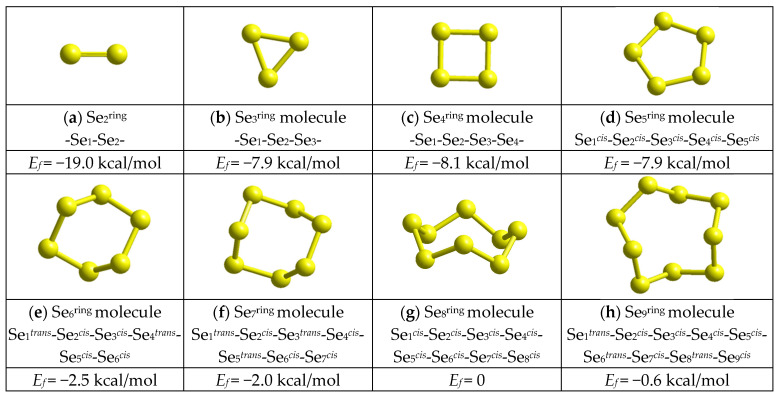
The optimized ball-and-stick presentation of ring-like MFCs Se_n_^ring^: Se_2_^ring^ (**a**), Se_3_^ring^ (**b**), Se_4_^ring^ (**c**), Se_5_^ring^ (**d**), Se_6_^ring^ (**e**), Se_7_^ring^ (**f**), Se_8_^ring^ (**g**), and Se_9_^ring^ (**h**). Configurations of Se atoms composing Se_n_^ring^ MFCs (yellow-colored) and the cluster-forming energies of these molecules *E_f_* (in respect to the energy of the Se_8_^ring^ molecule) are given below in cluster nomenclature. Super-script at the Se atom (*^trans^* or *^cis^*) indicates the conformation topology of the five-membered Se_5_ fragment centered at this atom.

The optimized configurations of ring-like Se_n_^ring^ MFCs (expanding in sizes from Se_2_^ring^ cluster, that is, a simple double Se=Se bond, to a Se_9_^ring^ cluster) composed of *n* atoms in the most favorable five-membered (*cis*- or *trans*-) topological configurations and differentiated by mean overall CFE (cluster-forming energy) *E*_f_ determined in respect to the Se_8_^ring^ molecule (*E*_f_ = −67.215 kcal/mol [14]), are presented in Figure 5.

Because of abnormal steric constraints in the low-sized Se_n_^ring^ MFCs with n = 3, 4, 5 (presumably, due to deviation in valence of the -Se-**Se**-Se- bond angle from an average value approaching ~105° [15,43]), these MFCs possess a very unfavorable *E*_f_~−8 kcal/mol. However, in transition to more extended molecular entities with n > 6, this tendency drastically changes. The Se_n_^ring^ structures based on these ring-like MFCs became quite competitive, showing growing *E*_f_ values from −2.5 kcal/mol for each Se_6_^ring^ cluster (see Figure 5e) to −0.6 kcal/mol for each Se_9_^ring^ cluster (Figure 5h), with an obvious jump in *E*_f_ for the cycloocta-selenium Se_8_^ring^ molecule (see Figure 5g). As shown in [14], this ring molecule of $\bar{8}$2m symmetry possesses unique crown-shaped topology because of eight Se-Se bonds with ~2.34 Å distances, valency bond angles close to ~105.6°, and dihedral angles for each Se atom averaged near ~101.5°, thereby approaching these parameters in the known crystalline analogues (such as monoclinic α-Se or β-Se [16,17,18,19]). In this Se_8_^ring^ molecule, each Se atom positioned within a five-membered -Se_5_- fragment (Se_1_-Se_2_-**Se_3_**-Se_4_-Se_5_) is considered in respect to the *equiplane* formed by three central Se atoms (Se_2_-**Se_3_**-Se_4_) that fits in a so-called *cis*-configurated topology where both terminated atoms (Se_1_ and Se_5_) are located in the same half-space (in respect to this Se_2_-**Se_3_**-Se_4_ plane). The overall CFE of this Se_8_^ring^ molecule (*E*_f_ = −67.215 kcal/mol [14]) is the best among all Se_n_ species, thus being suitable to normalize the CFE for other MFCs and NFCs. Noteworthy, other competitive MFCs Se_n_^ring^ with n > 6 reproduced in Figure 5 possess mixed *cis*-*trans*-configuration topology (thus, e.g., six Se atoms forming a ring-like Se_6_^ring^ molecule obey the *trans*-*cis*-*cis*-*trans*-*cis*-*cis* sequence of Se atoms Se_1_*^trans^*-Se_2_*^cis^*-Se_3_*^cis^*-Se_4_*^trans^*-Se_5_*^cis^*-Se_6_*^cis^*).

**Figure 6:**
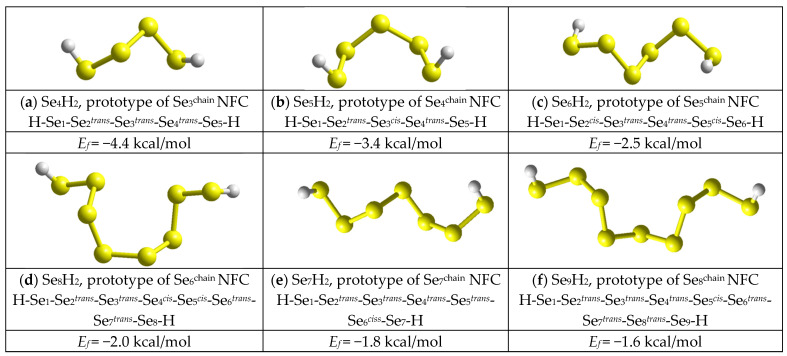
The optimized ball-and-stick presentation of the most favorable molecular prototypes of chain-like NFC Se_n_^chain^ derived from Se_n_^ring^ molecules by breaking in Se atom position: Se_3_^chain^ (**a**), Se_4_^chain^ (**b**), Se_5_^chain^ (**c**), Se_6_^chain^ (**d**), Se_7_^chain^ (**e**), and Se_8_^chain^ (**f**). Two-fold coordinated Se atoms and terminated H atoms are yellow- and grey-colored, respectively. Configurations of Se atoms within these molecules and CFE *E_f_* of Se_n_^chain^ NFC (in respect to the energy of Se_8_^ring^ molecule) are given below clusters nomenclature. Super-script (*^trans^* or *^cis^*) at the Se atom means conformation topology of five-membered fragment centered at this atom (the terminated H atoms are also included).

The iso-compositional chain-like Se_n_^chain^ NFCs can be derived from the Se_n_^ring^ MFCs depicted in Figure 5 by breaking in the respective Se atom position and saturation of the destructed bond by terminated hydrogen (H) atoms to form molecular prototypes of these NFCs compositionally equivalent to H_2_Se_n-2_ (see Figure 6), which are most suitable for further CINCA calculations [44,45].

**Figure 7:**
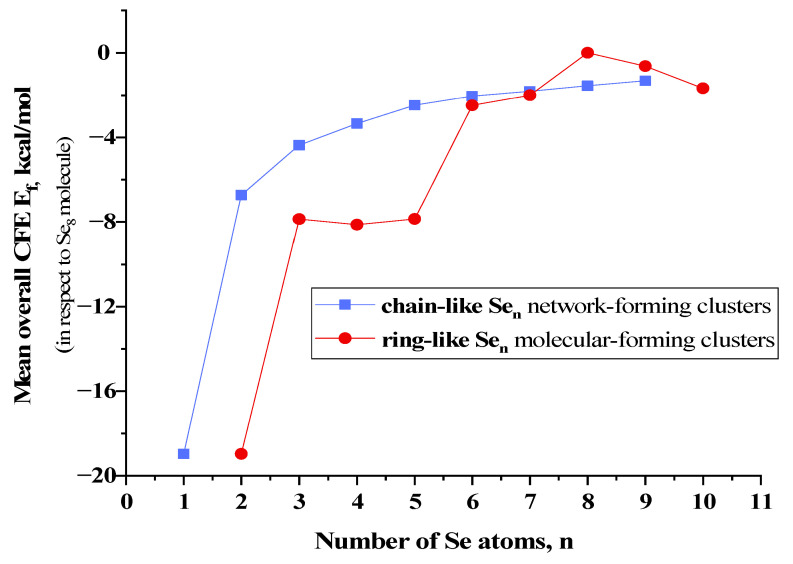
A comparison of mean overall cluster-forming energies E_f_ (determined in respect to the energy of Se_8_ molecule, −67.215 kcal/mol [14]) for Se_n_ clusters in chain- and ring-like conformations.

Under such chain-like Se_n_^chain^ architectures (see Figure 6), there are no any steric restrictions to stabilize self-consistent molecular forming configurations like in Se_n_^ring^ MFCs. As a result, the low-sized Se_n_^chain^ NFCs with n *=* 3, 4, 5 prevail by their CFE over Se_n_^ring^ MFCs, as this follows from the *E*_f_ comparison in Figure 7. Starting from n *=* 6, this slight growing trend in *E*_f_ energies remains nearly the same for both Se_n_^chain^ NFCs obeying mixed *cis*-*trans*-configuration topology and Se_n_^ring^ MFCs (apart from jump in CFE for Se_8_^ring^ MFCs). Of course, because of ignoration in inter-chain interactions, the Se_n_^chain^ NFCs keep full symmetry in the distribution of *cis*- and *trans*-configurated fragments (see Figure 6).

By returning to the disproportionality reaction (1), we come to the conclusion that all controversies between *cis*-configurated Se_8_^ring^ MFCs and *cis*-*trans*-configurated Se_8_^chain^ NFCs are expected in Se-rich As_x_Se_100−x_ alloys near some critical composition corresponding to As_7_Se_93_. Thus, a great number of atomic configurations consisting of an irregular sequence of randomly distributed *cis*- and *trans*-configurated five-membered Se linkages bridging cation-centered coordination polyhedrons (such as AsSe_3/2_ pyramidal units) which possess highly-deviated specific free energies are stabilized in a chain network of g-As_x_Se_100−x_ alloys at x < 7 undergoing nanostructurization without extractions of ring-like molecular entities, only due to changes in the fraction of these *cis*- and *trans*-configurated Se linkages. To activate such transformations (employing compositional-technological approaches), the energetic barrier between *cis*- and *trans*-configurated -Se_5_- linkages within Se_8_^ring^ MFCs and Se_n_^chain^ NFCs should be overcome. Accepting the respective CFE as given in Figure 5 and Figure 6, this barrier is estimated to be ~0.40 kcal/mol [14], the value which is quite comparable with the known energetic barrier of *ring*-to-*chain* transition in liquid Se [21,22].

The absence of a considerable amount of Se_8_^ring^ MFCs in the arsenoselenides is also proved from the DSC-TOPEM^®^ measurements for the g-As_5_Se_95_ alloy subjected to nanomilling [46]. Under *nanomilling*, a clear depression was found in the glass-transition temperature of this alloy, accompanied by slight increase in the heat capacity and enthalpy difference. From a microstructure perspective, such effects are rather typical for glasses not affected by changes in molecularity, instead of drastic glass-transition temperature increases in the over-stoichiometric arsenoselenides enriched in molecular entities [11,12,13].

## 3. Methods

### 3.1. Preparation and Characterization of Se-Rich Glassy Arsenoselenides As_x_Se_100−x_ (x < 10)

Samples of arsenoselenide alloys As_x_Se_100−x_ enriched in Se content (x < 10) were prepared from high-purity elemental precursors (As and Se of 5N purity) using a vibrational melt-quenching route as described in more detail elsewhere [11,12,13,14]. Sealed ampoules filled with As and Se in As_x_Se_100−x_ proportion were placed in a rocking furnace, heated to 650 °C, and homogenized. Then, they were placed vertically, cooled to 500 °C, and quenched in water. To eliminate the residual stresses possible in bulky ingots under rapid cooling, they were annealed at 10–15 °C below the glass-transition temperature *T*_g_.

The synthesized As_x_Se_100−x_ alloys were amorphous, as it follows from their XRD patterns (see, e.g., Figure 1) showing diffuse peak-halos typical for amorphous substances, conch-like fracture of the prepared cut sections, and IR transparency of glass bulks [14]. However, ‘pure’ Se samples (x = 0) prepared in these conditions were rather glassy-crystalline, since the sharp reflexes of the trigonal *t*-Se phase overlapped with ‘amorphous’ peak-halos were found in their XRD patterning (see, e.g., Figure 2). The values of mean interatomic spacing *d_s_^m^* in the prepared As_x_Se_100−x_ glasses (x < 10) calculated from their macroscopic densities (which were in a range of 4.32–4.26 g·cm^−3^) were 3.87–3.89 Å.

Mechanical milling was performed in a Pulverissete 6 mill operational under a protective Ar atmosphere and at 500 min^−1^ speed for 60 min in 250 mL tungsten carbide chamber loaded with 50 balls (10 mm in diameter) using ~3g of the alloy sieved under 200 μm. The energy transferring to the powder under these milling conditions was ~320 kJ/g [14]. Such high-energy mechanical milling ensures an effective activation of the examined alloys when employing a contemporary chalcogenide mechanochemistry platform [3].

### 3.2. Medium-Range Structure of Nanostructured Amorphous Alloys by the XRD Analysis

The XRD patterns of the amorphous arsenoselenide alloys were collected using a STOE STADI P diffractometer operated in transmission mode with Cu Kα_1_-radiation, a linear position detector, and curved Ge monochromator on a primary beam [11,12,13,14].

Preliminary processing of the XRD patterns in Se-rich arsenoselenides was performed using databases [47,48] related to monoclinic arsenic As_2_Se_3_ (the JCPDS card No. 65-2365) and known Se allotropes (the JCPDS card No. 73-0465 for trigonal *t*-Se, No. 71-0528 for monoclinic α-Se, and No. 73-2121 for monoclinic β-Se) [15,16,17,18,19,20]. Specifically, in identification of *t*-Se crystallites, we accepted into account the most pronounced Bragg-diffraction lines (with intensities above 10 %), which arise from a set of crystallographic planes corresponding to inter-planar distances *R*^100^ = 3.7812 Å (2*θ* = 23.509°, *I* = 43.9%), *R*^101^ = 3.0056 Å (2*θ* = 29.699°, *I* = 100%), *R*^110^ = 2.1831 Å (2*θ* = 41.323°, *I* = 16.7%), *R*^102^ = 2.0719 Å (2*θ* = 43.652°, *I* = 31.9%), *R*^111^ = 1.9977 Å (2*θ* = 45.361°, *I* = 22.4%), *R*^201^ = 1.7663 Å (2*θ* = 51.710°, *I* = 20.7%), *R*^112^ = 1.6377 Å (2*θ =* 56.114°, *I* = 11.1%), *R*^20 2^ = 1.5028 Å (2*θ* = 61.67°, *I* = 11.4%), *R*^210^ = 1.4292 Å (2*θ* = 65.229°, *I* = 14.6%), and *R*^123^ = 1.0806 Å (2*θ =* 90.932°, *I* = 10.7%).

The medium-range structure of the alloys was identified by the XRD analysis in application to diffuse peak-halos characteristic of amorphous substances; in part, the FSDP, which is a signature of structural entities forming IRO over a scale of a few tens of Å (reproduced near scattering vectors *Q*_1_ ≅ ~(1–1.5) Å^−1^) [31], and the SSDP (in the Elliott’s terminology [32]) or the principal diffraction peak (in terms of Zeidler and Salmon [33]), which is a signature of ERO observed near *Q*_2_~(1.9–2.2) Å^−1^. In the XRD patterning of As-Se alloys [11,12,13], the FSDP is revealed due to a diffuse peak-halo positioned at 2*θ*~(15–22)°, corresponding to real-space correlations commensurable with the intermediate scale of some network-forming species, while the SSDP, which is responsible for the sizes of these species close to mean interatomic spacing *d_s_^m^*, is revealed at 2*θ*~(28–33)° [31,32,33,34]. The three-peak structure of amorphous substances is completed by the TDP, which is the third of the principal diffuse peak-halos positioned near 2*θ*~(50–60)° (*viz*. *Q*_3_ ≅ ~(3.3–4.0) Å^−1^) revealed due to the shortest nearest-neighbor separation in glass approaching a few Å [33,34].

In glassy chalcogenides, another anomaly in the XRD patterning known as the pre-FSDP [29] appears at 2*θ*~(5–7)°, i.e., near *Q*~0.5 Å^−1^, where there are no any inter-planar correlations ascribed to the crystalline counterparts of these substances. The diffuse peak-halo in this domain is explained as arising from prolonged inter-atomic correlations *d_s_*~(15–20) Å [11,12,13,14,29]. In amorphous substances, this feature is unreproducible in the structure factor determination (like halos at higher 80–90° angles such as the FDP, the fourth diffraction peak). To date, no reproducible compositional changes have been detected for these peak-halos; nevertheless, their positioning should be accepted to avoid incomplete XRD patterning.

The XRD patterns were parameterized, accepting diffuse peak-halos in a glass as originated from superimposed inter-planar and inter-atomic correlations (see, [11,12,13,14]). The XRD profiles were processed with the STOE WinXPOW 3.03 [49] and PowderCell 2.4 programs [50], following normalization in respect to the maximum peak.

The angular position of the diffraction peak (2*θ*) and full width at half maximum (FWHM, Δ*W*) were defined with ±0.05°2*θ* accuracy, the scattering vector (*Q*) and width in a reciprocal space (Δ*Q*) being calculated as

*Q* = (4π/*λ*)⋅sin*θ*, (2)

Δ*Q* = (4π/*λ*)⋅sin(Δ*W*/2). (3)

The characteristic distance *R* (the spacing of diffuse peak-halo responsible quasi-periodicity) and correlation length *L* (the distance over which this quasi-periodicity is maintained) were defined as

*R* = 2π/*Q*, (4)

*L* = 2π/Δ*Q*. (5)

In application to amorphous arsenoselenides, we also explore the concept of diffuse peak-halos in their XRD patterns arising from coordination spheres (the inter-atomic distances) [51,52,53]), when the collected diffraction patterns are governed by the Ehrenfest relation [54]:

2*d_s_*·sin*θ* = 1.23·*λ*, (6)

where *d_s_* is the average inter-atomic distance between scattering centers (the radius of coordination sphere).

### 3.3. Cluster Modeling of Molecular-Network Conformations in Glassy Arsenoselenides

The geometrically optimized conformations of self-consistent molecular clusters and their network derivatives responsible for amorphization and reamorphization processes in Se-rich glassy arsenoselenides were identified, employing ab initio quantum-chemical modelling in terms of CINCA (the cation-interlinked network cluster approach) [44,45].

The chain-like Se_n_^chain^ NFCs were reconstructed from Se_n_^ring^ MFCs by breaking in the position of one Se atom followed by saturation of its parts by H atoms, thus stabilizing molecular H_2_Se_n−2_ = H-Se_1/2_…Se_1/2_-H prototypes. To account for specificity in the H-saturated bonding, the overall CFEs were corrected assuming self-termination in Se_2_^ring^ molecules. For convenience in comparison, the overall CFEs were averaged for all constituent atoms *E_f_* = (*E*_f_^Σ^)^av^ and recalculated in respect to the energy of the Se_8_^ring^ molecule (the basic unit in α/β-Se [15,16,17,18,19,20]. Only intra-chain interactions were taken into account under such modelling, ignoring inter-chain interactions in realistic molecular and network structures.

The HyperChem Release 7.5 program package based on the restricted Hartree–Fock self-consistent field method with a split-valence double-zeta basis set and single polarization function 6-311G* [55,56] was employed to calculate the CFE (*E_f_*). Optimization and single-point energy calculations for self-consistent molecular clusters were performed using the Fletcher–Reeves conjugate gradient method until the root-mean-square gradient of 0.1 kcal/(Å·mol) was reached. Finally, the calculated *E_f_* energies were corrected on the energy of terminated H atoms transforming the chain-type NFC structures into quasi-molecular ones according to the algorithm developed elsewhere [57,58].

## 4. Conclusions

The paradigm of molecular-network conformations in Se-rich glassy arsenoselenides As_x_Se_100−x_ compositionally approaching pure Se (x < 10) is considered, employing XRD analysis of diffuse peak-halos and nanocrystalline reflections arising from Se polymorphs in their XRD patterning. Within a modified microcrystalline model, the changes with growing Se content in these alloys are interpreted in terms of suppression in an intermediate range ordering due to shifts to high diffraction angles and a narrowing of the FSDP (the first sharp diffraction peak)-related diffuse peak-halo, accompanied by enhancement in extended range ordering due to shifts to low diffraction angles and a broadening of the SSDP (the second sharp diffraction peak)-related diffuse peak-halo. Overlapping of these peak-halos is enhanced in glassy arsenoselenides approaching Se, tending to a unified FSDP-SSDP-related diffuse peak-halo with characteristic doublet asymmetry due to the nanocrystalline remnants of trigonal *t*-Se.

Enhancement of crystallization processes related to *t*-Se phase is a principal feature of nanomilling-driven effects in Se-rich arsenoselenides, the nanostructurization response in these alloys being revealed as an enhancement in their intermediate range ordering (due to a slightly high-angular shifted but essentially broadened FSDP-related peak-halo), counterbalanced by depression in their extended range ordering (due to a high-angular shifted but essentially narrowed SSDP-related peak-halo), meaning fragmentation impact on the correlation length of the FSDP-responsible entities, accompanied by an agglomeration impact on the correlation length of the SSDP-responsible entities. The FSDP- and SSDP-related diffuse peak-halos become more distinguishable in the XRD patterns of nanostructured arsenoselenides, being associated with other contributions from crystalline remnants as those expected in transition to alloys with higher Se content.

In arsenoselenides possessing a chain-type network composed of *cis*- and *trans*-configurated multiatomic Se fragments bridging coordination polyhedra such as AsSe_3/2_, nanomilling-driven heat-transfer phenomena are defined by ring-to-chain transitions resulting in a minor *trans*-configuration-enhanced decrease in their glass transition temperatures. The irregular sequence of randomly distributed *cis*- and *trans*-configurated Se linkages in the examined Se-rich arsenoselenides is visualized by ab initio quantum-chemical modeling of Se_n_ chain- and ring-like conformations. The critical point of molecular-network disproportionality analysis in As_x_Se_100−x_ alloys obeying the chain-crossing model corresponds to x = 7, serving as an equilibrium between mixed *cis*-*trans*-configurated Se_7_ chains and exceptionally *cis*-configurated Se_8_ rings.

At the basis of the developed models, the paradigm of thermodynamically stable molecular-network conformations in nanostructured Se-rich arsenoselenides As_x_Se_100−x_ (x < 10) is surely resolved in favor of chain-like network-forming conformations built of preferentially *trans*-configurated Se fragments.

## Data Availability

The original contributions presented in this study are included in the article. Further inquiries can be directed to the corresponding author.
